# UYSD: a novel data repository accessible via public website for worldwide population frequencies of Y-SNP haplogroups

**DOI:** 10.1038/s41431-025-01854-5

**Published:** 2025-05-08

**Authors:** Arwin Ralf, Dion Zandstra, Bram van Wersch, Zehra Köksal, Maarten H. D. Larmuseau, Alexandra Rosa, Mark A. Jobling, Maria E. D’Amato, Cornelius Courts, Mario Gysi, Cordula Haas, Rodrigo Flores, Maximilian Neis, Jon H. Wetton, Kevin Kiesler, Adam Ameur, Simon Azonbakin, Alexandra Bôžiková, Andrej Choma, Maria Corazon De Ungria, Beatrice Corradini, Catarina Cruz, Bettina Dunkelmann, Gianmarco Ferri, Jan Fleckhaus, Domniki Fragou, Noah Gaens, Rita Gonçalves, Dubravka Havaš Auguštin, Katharina Helm, Petra Hölzl-Müller, Michał Kaliszan, Mohaimin Kasu, Leda Kovatsi, Mpasi Lesaoana, Natsuko Mizuno, Franz Neuhuber, Jana Nováčková, Alena Ňuňuková, Horolma Pamjav, Walther Parson, Yerlan Ramankulov, Héctor Rangel Villalobos, Krzysztof Rębała, Siiri Rootsi, Jazelyn Salvador, Jelena Šarac, Carolyn R. Steffen, Vlastimil Stenzl, Tibor Török, Richard Villems, Haruhiko Watahiki, Maxat Zhabagin, Peter M. Schneider, Manfred Kayser

**Affiliations:** 1 Department of Genetic Identification, Erasmus MC University Medical Center Rotterdam, Rotterdam, the Netherlands; 2 Laboratory of Human Genetic Genealogy, Department Human Genetics, KU Leuven, Leuven, Belgium; 3 University of Madeira, Funchal, Portugal; 4 University of Leicester, Leicester, United Kingdom; 5 University of the Western Cape, Western Cape, South Africa; 6 University Hospital of Cologne, Institute of Legal Medicine, Cologne, Germany; 7 University of Zurich, Zurich, Switzerland; 8 University of Tartu, Tartu, Estonia; 9 National Institute of Standards and Technology, Gaithersburg, MD USA; 10 Department of Immunology, Genetics and Pathology, Uppsala University, Uppsala, Sweden; 11 SciLifeLab, Uppsala University, Uppsala, Sweden; 12 Institute of Forensic Science, Bratislava, Slovak Republic; 13 University of the Philippines Diliman, Quezon City, Philippines; 14 Institute of Legal Medicine, University of Modena and Reggio Emilia, Modena, Italy; 15 University of Salzburg, Salzburg, Austria; 16 Ludwig Maximilian University, Institute of Legal Medicine, Munich, Germany; 17 Aristotle University of Thessaloniki, Thessaloniki, Greece; 18 Institute for Anthropological Research, Zagreb, Croatia; 19 Institute of Legal Medicine, Medical University of Innsbruck, Innsbruck, Austria; 20 Medical University of Gdansk, Gdańsk, Poland; 21 Lesotho Mounted Police, Maseru, Lesotho; 22 National Research Institute of Police Science, Kashiwa, Japan; 23 Institute of Criminalistics, Prague, Czech Republic; 24 Hungarian Institute for Forensic Sciences, Institute of Forensic Genetics, Budapest, Hungary; 25 Forensic Science Program, The Pennsylvania State University, University Park, PA USA; 26 Nazarbayev University, Astana, Kazakhstan; 27 University of Guadalajara, Guadalajara, Mexico; 28 University of Szeged, Szeged, Hungary; 29 National Center for Biotechnology, Astana, Kazakhstan; 30 Present Address: Department of Pathology and Clinical Bioinformatics, Erasmus MC University Medical Center Rotterdam, Rotterdam, the Netherlands

**Keywords:** Genetic databases, Genetic variation

## Abstract

For decades, there has been scientific interest in the variation and geographic distribution of paternal lineages associated with the human Y chromosome. However, the relevant data have been dispersed across numerous publications, making it difficult to consolidate. Additionally, understanding the relationships between different variants, and the tools used to analyze them, have evolved over time, further complicating efforts to harmonize this information. The Universal Y-SNP Database (UYSD) marks a substantial advancement by providing a comprehensive and accessible platform for Y-SNP and haplogroup data from populations around the world. UYSD harmonizes diverse datasets into a unified repository, facilitating the exploration of global Y-chromosomal variation. The platform handles data generated with both high- and low-throughput technology and is compatible with the automated analysis software tool, Yleaf v3. Key functionalities include the ability to: i) visualize haplogroup distributions on an interactive world map, ii) estimate haplogroup frequencies in geographic regions with sparse data through interpolation, and iii) display detailed phylogenetic trees of Y-chromosomal haplogroups. Currently, UYSD encompasses data from over 6,600 males across 27 populations. This dataset largely aligns with known global Y-haplogroup patterns, but also reveals unexplored finer-scale geographic variations. While the present dataset is largely European-centered, UYSD is designed for ongoing expansion by the scientific community, aiming to include more global data and higher-resolution population sequencing data. The platform thus offers valuable insights into human genetic diversity and migration patterns, serving several fields of research such as: human population genetics, genetic anthropology, ancient DNA analysis and forensic genetics.

## Introduction

Single nucleotide polymorphisms located in the male-specific region of the human Y chromosome (Y-SNPs) escape reassortment through crossover, thus defining haplotypes (which can be grouped together as ‘haplogroups’) which form a robust phylogeny. Y-SNPs have been the subject of research for nearly 40 years [1, 2]. By the end of the last century, typically only a dozen or fewer Y-SNPs were analyzed due to limitations in DNA technology and knowledge of markers [3, 4]. Yet, as evident from a plethora of Y-SNP-based population studies since the early 2000s, there was a great interest in understanding human evolution and migration history through the lens of the Y chromosome. In the early days of Y-haplogrouping, most studies focused on either the most basal Y-haplogroups [5–8], or used a hierarchical approach to attain higher resolution Y-haplogroup inference [9–12]. A pivotal moment for Y-SNP research was the publication by Karafet et al. in 2008 [13], in which the previously known Y-chromosomal haplogroup tree was thoroughly revised by incorporating hundreds of newly discovered Y-SNPs. More recently, the number of known Y-SNPs increased dramatically as a result of the application of second-generation DNA sequencing technologies, allowing the sequencing of more individuals and more parts of the Y chromosome.

Second-generation sequencing technologies led to the development of larger targeted genotyping assays to analyze hundreds of Y-SNPs simultaneously, compared to the dozens that could be analyzed before using, e.g., Sanger sequencing or minisequencing [14–17]. Additionally, whole-genome sequencing (WGS) data can now be used to obtain very highly-resolved Y-haplogroups [18]. An example of the capabilities offered by WGS can be found in the study published by Hallast et al. in 2015 [19], where instead of several hundreds, over 13,000 Y-SNPs had been incorporated into a single Y tree. As the number of available Y-SNPs increased, the need for automated analysis methods to determine Y-haplogroups from bulk data also grew, resulting in the development of several bioinformatic tools with that specific purpose [20–22]. Currently, the most extensive Y chromosome haplogroup trees contain hundreds of thousands of Y-SNPs and differentiate tens of thousands of Y-SNP-based haplogroups, for example, YFull’s YTree v13.01.00 contains over 400,000 Y-SNPs and defines over 60,000 distinct haplogroups [23]. These numbers are expected to rise further, in particular through in-depth analysis of Y chromosomes from more diverse populations.

However, the rapid increase in the number of identified Y-SNPs created challenges in harmonizing the results of various studies and reconciling competing nomenclature systems. To address these issues, van Oven et al. proposed a minimal reference phylogeny for the human Y chromosome [24]. Although the criteria for including haplogroups were objective, the depth of the phylogeny was constrained by the available data, which was unevenly distributed across haplogroups and populations. A key limitation of this approach was the absence of a centralized database for Y-SNP haplogroups, which could provide consistent haplogroup frequency data across different populations. The lack of a broadly used data repository for Y-SNPs contrasts with the situation for autosomal STRs, Y-STRs, and mitochondrial DNA, for which population frequency databases have already been in use for many years now, such as STRidER [25], YHRD [26], and EMPOP [27]. While YHRD does allow the storage of Y-SNP data, it is limited to the minimal reference phylogeny and primarily oriented toward forensic applications [28]. Currently, most of the published Y-SNP data are scattered across many different publications, which requires a researcher to collect and compile them individually. Moreover, different studies have often used different haplogrouping methods and interrogated different Y-SNPs, rendering the necessary manual harmonization of the results error-prone, highly laborious, and sometimes impossible.

To overcome previous shortcomings, we here introduce the Universal Y-SNP Database (UYSD), a centralized data repository developed to consolidate and harmonize Y-SNP data, along with a public website (https://ysnp.erasmusmc.nl) that allows researchers to access these data for various research purposes and to submit their own Y-SNP data. The data repository was designed to be independent of Y-SNP genotyping technology, making it adaptable for both historical Y-SNP data, generated by former DNA technologies with limited markers, and bulk data produced by modern technologies that handle a large number of markers. To achieve this, the data submission system is made compatible with Yleaf version 3 [22], a tool that had previously been developed and further updated for Y-haplogrouping. As a first attempt to populate the database, we generated a partly de novo dataset as part of a multicenter study on Y-SNPs, which was complemented by samples from previous studies. This resulted in a total dataset of 6637 male individuals from 27 different countries. We anticipate that UYSD will become a highly valuable platform for sharing Y-SNP haplogroup data across various fields of human genetics. We hope that it will encourage the Y chromosome research community to contribute their Y-SNP haplogroup data, thereby expanding the database and enhancing its utility for addressing diverse research questions.

## Materials and methods

### Platform development and features

The UYSD website was developed using the Django web framework v4.1.4 [29], which is built on Python [30], for both back-end and front-end tasks. The back-end of the platform is supported by an SQLite database v3.45.0 [31]. The database schema was designed to store data about user authentication, haplogroup, Y-SNP, geographic region, and sample data. The platform is based on the YFull tree version 10.01 [23], and at the time of writing considers 28,379 unique branches. As Y-haplogroup trees are constantly being expanded and refined, the underlying tree will be periodically updated in the future, these updates will be synchronized with updates of the phylogenetic tree used by Yleaf. The world map is based on Leaflet v1.8.0 [32] and uses OpenStreetMap API [33] map tiles collected from MapTiles API [34]. Shape files for the country and region data were collected in GeoJSON format from Natural Earth [35].

UYSD is compatible with Yleaf v3 [22], meaning that researchers can readily upload their output files from Yleaf to the platform. Alternatively, for compatibility with pre-second-generation sequencing data, it is possible for researchers to manually submit a list of genotyped Y-SNPs together with the haplogroups assignment for each sample. All data submissions are automatically checked for incorrect formatting and validity issues of the data (e.g., duplications of sample names, incorrect spelling of country/region names, or missing files). If errors are detected, appropriate messages guide users in correcting their submissions.

Users can perform searches on UYSD by haplogroup or Y-SNP name including synonyms of equivalent Y-SNPs. For example, haplogroup R-L51 can be reached by entering the full haplogroup name *R-L51*, or just the Y-SNP name *L51*, alternatively searching for *M412* (a synonym), or entering *Y410* (an equivalent Y-SNP) will yield the same result. The system processes all sample data to count occurrences of derived and ancestral alleles for available Y-SNP (including equivalent Y-SNPs) across geographic regions and generates a world map. Heat map percentages on the interactive world map show the proportion of samples belonging to the queried haplogroup in each region, calculated by dividing the number of samples with the derived allele by the total number of samples analyzed for that area in which at least one Y-SNP that defines the queried haplogroup was analyzed. It is possible to show the Y-haplogroup frequencies on a scale from 0 to 100%; however, as some haplogroups are rare, their population-specific frequencies can also be visualized relative to the population with the highest observed frequency. Detailed information about samples relevant to the query, such as subpopulation assignments or available Y subhaplogroup typings, can be accessed by clicking on a specific country on the map. For large countries (e.g., Russia or China), it may be more informative to provide or view frequencies at a sub-country regional level rather than at the national level – an option also implemented in UYSD.

The filtering option is intended to narrow down haplogroups using a ‘*’ symbol. When a user enters a query including a *, the input is split between the main haplogroup and the filtering criterion. For example, E*(xV13) will show the regional frequencies based on all samples belonging to haplogroup E, except for those belonging to E-V13. Each specified haplogroup and filtering criterion is validated. If all are valid, the filtered haplogroups are used to generate the map data, otherwise a warning is returned to the user.

If the user enables the interpolation option, haplogroup frequencies for geographic regions without data are estimated, in case there are sufficient data from surrounding regions. For each region, nearby regions with sufficient data (i.e., at least 50 individuals) are identified, and a weighted average of haplogroup frequencies is calculated using the inverse distances between the regions, applying the Haversine formula to find the shortest spherical distance between their borders. Interpolation proceeds only if there are at least three surrounding regions with frequency data within 1000 km of the region to be interpolated.

Lastly, UYSD also allows displaying the full phylogenetic tree based on all Y-SNP and haplogroups included in the database, or by searching for specific Y-SNPs or samples through queries on three separate tabs.

A user manual further describing the different functions that UYSD offers can be found in the Supplementary Text and on the UYSD website.

### Usage conditions

Although UYSD was developed as a tool for the academic community, information about the distribution of Y-haplogroups may also be of interest to a broader public—for example, persons who have undergone Y-chromosomal analysis through direct-to-consumer DNA testing companies or citizen scientist. Therefore, UYSD usage is not restricted, and no user registration is required for access. However, to ensure quality control and prevent data contamination, only researchers with institutional email addresses can create accounts and submit data. While we recognize the valuable contributions of citizen scientists and acknowledge that this restriction may limit UYSD’s growth, maintaining the integrity of the database is paramount. Restricting data submission to academic researchers helps minimize errors, ensure accountability, and maintain high data quality. Moreover, only Y-SNP and Y-haplogroup population data that have already been published in a peer-reviewed scientific journal are eligible for submission. As part of the submission process, the data-submitting user is asked to provide the reference for their data and the platform will retain the link with this publication. By keeping this direct link to the original publications, it is possible to obtain more information about a given Y-haplogroup dataset from the original paper than can be accessed via the UYSD.

By only allowing published data to be submitted to UYSD, we aim to ensure that the data in the database adhere to scientific and ethical principles, assuming that these standards have been met in the original peer-reviewed publication. Only when compelling evidence of scientific misconduct is presented to the database administrator by a third party, can the administrator decide to remove the respective dataset from the platform. In such case, the administrator will discuss the concerns with the user who submitted the data and may consult the scientific journal that had originally published that work. Data of publications that are retracted because of ethical concerns will immediately be removed once the administrator is notified. Ultimately, the user that submits the data to UYSD remains fully responsible for the data and can be directly contacted through a form on the platform. Before submitting data to the platform, it is mandatory to comply to the aforementioned terms. UYSD users that utilize information obtained via the database for their publications are urged to cite both the original publication as well as UYSD (i.e., the present paper).

### DNA samples

In the context of a collaborative multicenter study, a total of 29 institutes contributed to the initial UYSD dataset. Y-haplogroups were assigned to a total of 6637 males from 27 worldwide populations, including countries from Europe, Africa, Asia, and America. Some of the data were generated using samples from older DNA-collections and were therefore collected without ethical review or written informed consent. In this context, it is important to emphasize that these samples are not associated with any personal information and that Y-haplogroups, by definition, do not enable individual identification. Supplementary Table 1 provides more details on each of the population cohorts included in this study.

### Genotyping

The majority of the samples (78%) currently included in UYSD were genotyped de novo using the *Ion AmpliSeq™ HID Y-SNP Research Panel v1* (Thermo Fisher Scientific, Waltham, Massachusetts, United States) targeting over 1500 Y-SNPs allowing the classification of approximately 1000 Y-haplogroups [15]. Further, 328 samples (5%) included in UYSD were previously typed with minisequencing (SNaPshot) assays targeting no more than a few dozen Y-SNPs. Lastly, 1145 samples (17%) from three populations were previously analyzed with non-targeted WGS, resulting in tens of thousands of Y-SNPs being analyzed. For two WGS datasets, a complete analysis was performed using all Y-SNPs available through Yleaf v3. For the third WGS dataset, in line with previous agreements, only 772 Y-SNPs that overlapped with those covered by the *Ion AmpliSeq™ HID Y-SNP Research Panel v1* were analyzed.

### Comparative data analysis

Since the samples in this study were analyzed using different DNA technologies, each examining varying numbers of Y-SNPs and classifying Y-haplogroups at different levels of resolution, we inferred a reduced phylogenetic tree to enable comparative analysis between the population samples. To qualify for inclusion in this tree, each Y-SNP had to be typed in at least 90% of the samples. Additionally, each included Y-SNP was required to have a frequency of at least 5% in one of the analyzed populations. A total of 188 Y-SNPs met these criteria and were, therefore, included in comparative analyses between the 27 study populations. Arlequin v3.5 [36] was used to compute population pairwise *F*_ST_ based on the haplogroup frequencies of the 188 Y-SNPs. Nei’s gene diversity was calculated for both the reduced set of 188 Y-SNPs and for the full set of Y-SNPs available for each population [37].

## Results

### Worldwide distributions of basal Y-haplogroups

**Fig. 1:**
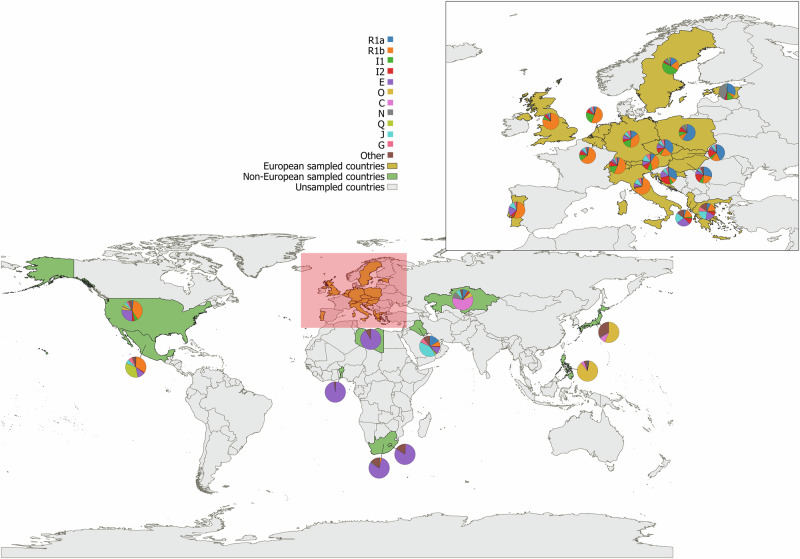
Worldwide distribution of basal Y-haplogroups in the 27 populations currently included in UYSD. The main figure shows the whole world while the panel shows the European data specifically. The figure was made using QGIS and only the most frequently observed basal haplogroups are assigned a color in the pie charts.

Although the data currently provided to the UYSD were skewed towards European populations, some typical global geographic patterns were observed [38]. For example, as evident in Fig. 1, haplogroup O is prevalent in East Asia, haplogroup R in Europe, haplogroup C in Central Asia, and haplogroup E in Africa. Within Europe, R1b is more frequent in Western Europe [39], while R1a is more prevalent in the East [40], haplogroup I1 is found more often in Northern Europe, and I2 and J are more common in Southern Europe (Fig. 1) [41, 42]. The most frequently observed haplogroup in this study was R (39%), followed by E (16%) and I (15%). The predominance of European populations in this study (60% of all samples) can explain the high prevalence of haplogroup R and I. Although haplogroup E has the highest frequencies on the African continent, some of its subclades are also commonly found in Europe, with the highest frequencies in the southern regions (Fig. 1) [42]. The Y-haplogroup variation in the United States and Mexico stands out, as reflected in the many non-native haplogroups that were found in addition to the native haplogroup Q [43]. This can be attributed to the history of the American continents being shaped by migration from Europe, Africa, and Asia.

### Worldwide distribution of higher-resolution Y-haplogroups

**Fig. 2:**
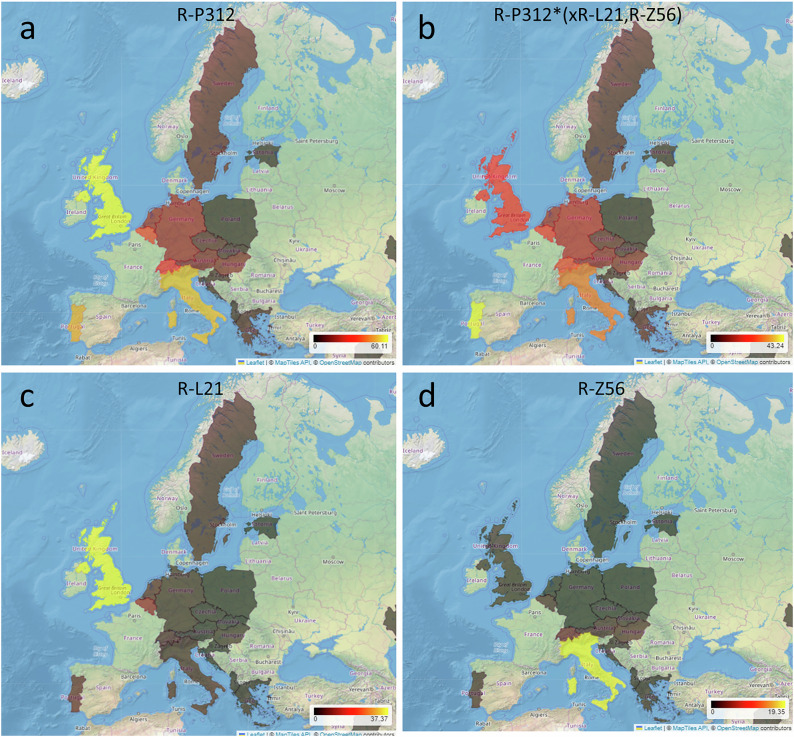
Screenshots from the UYSD website using the Haplogroup map function for Y-haplogroup R-P312 in Europe. **a** illustrates the geographic frequency distributions of haplogroup R-P312 including all of its subgroups, while **b**–**d** show specific subgroups within R-P312. Only countries with records for the haplogroup of interest are shaded.

Figure 2 provides an illustrative example of four higher-resolution lineages, namely the monophyletic R-P312, R-L21, and R-Z56, and the paraphyletic R-P312*(xR-L21, R-Z56). Figure 2a shows the prevalence of Y-haplogroup R-P312 in Europe, with the highest frequency in the United Kingdom (~60%). Figure 2b depicts the distribution of the paraphyletic R-P312*(xR-L21, R-Z56), i.e., R-P312 excluding subgroups R-L21 and R-Z56, revealing its highest prevalence in Portugal (43%). When focusing on R-L21 alone (Fig. 2c), there is a high frequency in the United Kingdom (~37%), whereas it is rare elsewhere. In contrast, R-Z56 (Fig. 2d) is common in Italy (~19%), but rare in the other European populations analyzed in this study.

Notably, the most commonly observed higher-resolution Y-haplogroup in the whole dataset is E-V13, with a total of 228 observations and a frequency peak in Southeastern Europe (i.e., the Balkan area). This clade not only appears frequently but is also very widespread as observed before [44], being observed across 20 of the 27 populations analyzed. The second most frequent is E-U174 with 226 observations, but only in five, primarily African populations with frequencies of >30% in both West African Benin and Southern Africa. Other examples of frequently observed clades were R-CTS3402 with a predominantly Eastern European distribution and N-L1025 with a Northeastern European distribution.

A total of 902 different phylogenetic lineages were observed among the study participants, with 493 (55%) of these observed only once. Notably, 408 (83%) of these singletons were found in one of the two populations for which whole-genome sequencing (WGS) data were fully analyzed, supporting the high resolution achieved by fine-scale genotyping. In this study, data from two populations with available WGS (Belgium [45] and Sweden [46]) were analyzed and included in UYSD, significantly increasing the number of Y-SNPs and detected haplogroups compared to other genotyping methods (Table 1). On the other end of the spectrum stand population samples from Japan [47], Libya [48], and Benin [49], which were typed with only 10, 32, and 40 Y-SNPs, respectively.

**Table 1:** Basic characteristics of the 27 study populations (ordered from low to high gene diversity of the reduced haplogroups).

		Reduced haplogroups			Full haplogroups		
Population	Number of individuals	Gene Diversity	No. of unique hgs	Most frequent haplogroup	Gene Diversity	No. of unique hgs	Most frequent haplogroup
Libya	47	0.5749	4	E-M81 (49%)	0.5749	4	E-M81 (49%)
Kazakhstan	185	0.7284	30	C-L1373 (50%)	0.7317	38	C-L1373 (50%)
Benin	289	0.8078	14	E-U174 (30%)	0.8080	17	E-U174 (30%)
Philippines	189	0.8183	20	O-P164 (31%)	0.8714	31	O-Y14027 (25%)
South Africa	96	0.8322	16	E-U174 (33%)	0.8355	17	E-U174 (33%)
Lesotho	93	0.8334	12	E-U174 (31%)	0.8366	14	E-U174 (31%)
United Kingdom	190	0.84	38	R-DF13 (37%)	0.9468	67	R-DF13 (18%)
Croatia	185	0.8465	27	R-CTS1211 (26%)	0.8746	35	I-L621 (23%)
Poland	187	0.8546	37	R-M458 (32%)	0.9040	44	R-M458 (23%)
Albania	188	0.8642	32	E-V13 (26%)	0.8650	41	E-V13 (26%)
Portugal	191	0.8684	46	R-P312 (34%)	0.8877	69	R-P312 (31%)
Mexico	169	0.8753	39	Q-M3 (31%)	0.8867	58	Q-M3 (30%)
Japan	353	0.8862	19	O-K10 (20%)	0.8940	29	O-K10 (20%)
Slovakia	192	0.8875	39	R-CTS1211 (23%)	0.9201	57	R-CTS3402 (16%)
Estonia	400	0.8939	40	N-L550 (23%)	0.9212	57	N-L1025 (19%)
Czech Republic	182	0.9221	43	R-M458 (20%)	0.9404	59	R-M458 (18%)
Hungary	191	0.9308	43	I-L621 (15%)	0.9484	63	I-L621 (15%)
Italy	186	0.9362	44	R-BY1823 (13%)	0.9472	59	R-BY1823 (13%)
Greece	187	0.9436	42	E-V13 (14%)	0.9548	62	E-V13 (14%)
Sweden	475	0.9518	51	R-Z284 (10%)	0.9968	376	I-Y4115 (1%)
Belgium	270	0.9562	55	R-P312 (11%)	0.9934	209	R-DF98 (3%)
Netherlands	192	0.9568	42	R-Z30 (9%)	0.9717	70	R-Z30 (8%)
Iraq	192	0.9585	43	R-Z2124 (7%)	0.9681	60	R-M12149 (6%)
Switzerland	189	0.9612	44	R-L2 (9%)	0.9748	68	R-L2 (7%)
Germany	189	0.964	54	R-CTS1211/R-Z30 (7%)	0.9772	89	R-CTS1211 (6%)
Austria	380	0.9663	57	R-L2/E-V13 (6%)	0.9773	101	E-V13 (6%)
United States	1050	0.9665	111	R-DF13 (10%)	0.9816	211	E-U174 (7%)

### Population differences of Y-haplogroups

**Fig. 3:**
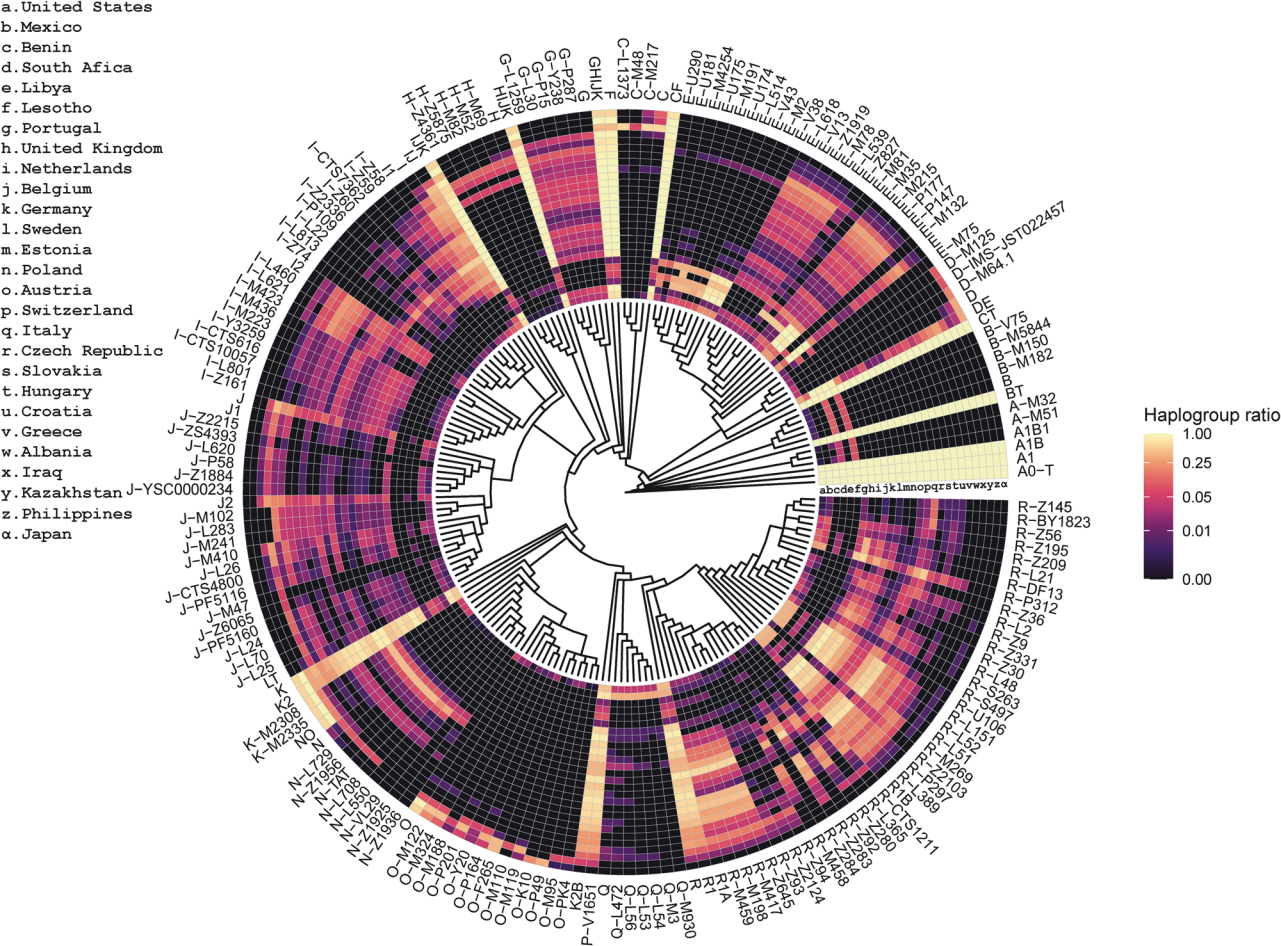
Visualization of the population frequencies of a subset of 188 Y-haplogroups available from all 27 study populations. A linearized visualization of these data can be found in Supplementary Fig. 1.

As previously mentioned, the 27 populations in this study were not all genotyped using the same DNA technology, resulting in varying numbers of Y-SNPs across samples, which is typical in Y-chromosomal population studies. Taking these differences into account, a reduced set of 188 Y-haplogroups was used for comparative population analyses. The phylogenetic tree and frequencies of these Y-haplogroups in each of the 27 study populations are shown in Fig. 3. Not surprisingly, the largest differences were observed between populations from different continents. Nevertheless, subtler differences could also be found in populations of the same continent. For example, in two East Eurasian populations here included, D subhaplogroups were commonly observed in Japan (33%), while they were rare (3%) in the Philippines. Haplogroup O was the dominant haplogroup in both populations (56% in Japan and 84% in the Philippines), yet major population differences were found at deeper subhaplogroup levels. For instance, the O-P164 lineage was found in >30% of the Filipinos but in less than 9% of the Japanese. In contrast, haplogroup O-K10 was observed in >30% of the Japanese, while being absent from the Philippines dataset. Differences could also be found when comparing the Y chromosome phylogenetic lineages between the studied African populations. While the combined frequency of haplogroups A and B is ~15% in South Africa and Lesotho, it is just over 1% in Benin. Similarly, E-U181 is found in ~10% of South African and Lesotho males but was not found in Benin. Conversely, E-M132 is absent in our South Africa and Lesotho sample set, while having a frequency of ~7.5% in Benin.

Given the European-focused nature of our dataset, we found it relevant to deepen the analysis in these population samples. Supplementary Fig. 2 shows an MDS plot based on the *F*_ST_ values estimated from Y-SNP haplogroup frequencies between the 17 European populations (for pairwise *F*_ST_ of all populations, including the non-European, see Supplementary Table 2). Notably, the first two dimensions together explain over 77% of the variation. There appears to be an East to West gradient moving from right to left in the first dimension of the MDS plot. The North-South distribution of the population samples is not clearly reflected in the MDS analysis. Neighboring countries with historical connections, such as Germany and Austria or the Netherlands and Belgium, often cluster together due to similar haplogroup compositions. However, Slovakia and the Czech Republic, despite their close proximity, do not cluster closely (Supplementary Fig. 2). A notable difference is the prevalence of Y-haplogroup R-CTS1211: it occurs in 24% of Slovakians (similar to Poland and Croatia, with 21 and 26%, respectively), but in less than 15% of Czechs (comparable to Hungary, where it is also 15%). This difference, combined with other less pronounced differences in haplogroup composition, may explain why the two do not cluster more tightly together in this analysis.

## Discussion

### UYSD technical performance

UYSD is designed as a repository for both low- and high-throughput Y-SNP haplogroup data, stored in a harmonized format and made publicly accessible for diverse research purposes. It aims to connect researchers interested in Y-chromosomal variation, supporting fields such as forensic genetics, human population studies, archaeogenetics, and genetic genealogy. By harmonizing various Y-SNP datasets using a consistent underlying phylogenetic Y chromosome tree in an automated manner, the platform allows researchers to easily relate their own data to previously published work. In this study, we demonstrated that the platform successfully achieves this by incorporating datasets from three different throughput levels.

There are several phylogenetic trees in use. In addition to the tree developed by YFull (utilized here), a commonly referenced tree is maintained by the International Society of Genetic Genealogy (ISOGG). However, ISOGG’s Y-DNA Haplogroup Tree has not been updated since 2020 and relied heavily on manual updating, which can introduce errors. Another option is the Y-DNA Haplotree developed by FamilyTreeDNA. While extensive, it lacks traceability—there are no version numbers, and changes are not publicly documented. In contrast, YFull’s YTree offers regular updates and comprehensive traceability, making it the preferred foundation for UYSD, at present. A disadvantage of the approach is that when data is stored in UYSD only variations which are incorporated in the underlying phylogenetic tree are stored. Any variants considered by alternative phylogenetic trees, private mutations, or variations that are not yet incorporated in the phylogenetic tree will not be stored. Consequently, if the phylogenetic tree used by UYSD is updated, newly added genetic variants (compared to the previous version) cannot be analyzed for samples that were included under the prior version.

To estimate the frequencies of Y-haplogroups in regions not yet covered by UYSD, interpolation can be used, provided there is sufficient data from surrounding areas. This method allows for an estimation of genetic patterns; however, it is important to recognize its limitations. Notably, interpolation does not account for natural barriers, such as mountains, oceans, or dense forests, which can restrict gene flow between populations. As a result, this approach may be less accurate in geographically close regions that are separated by significant natural obstacles. Additionally, historical, cultural, or political factors—unrelated to geographic proximity—may have played a crucial role in shaping the Y chromosome haplogroup composition of a given region. Events such as migrations, wars, trade routes, or isolation due to sociopolitical divisions can all influence genetic patterns in ways that simple geographic interpolation cannot capture. Thus, interpolation results should at best be considered as preliminary estimations and ideally should be confirmed by empirical data.

### Opportunities for future population studies

The basal Y-haplogroups, representing the deepest branches of the phylogenetic tree, originated tens of thousands of years ago and are now carried by millions of men worldwide, often exhibiting distinct geographical frequency distributions [38]. Within Europe, the strong differences in basal Y-haplogroup distributions observed here align well with previous literature (e.g., refs. [39–42, 50]). In contrast, our understanding of the geographic distribution of higher-resolution Y-haplogroups is more limited. These fine-scale phylogenetic clades have emerged more recently—on the order of thousands, rather than tens of thousands, of years ago [18]—resulting in their lower prevalence in present-day human populations and often more geographically restricted distributions. However, as these lineages become more finely divided, their frequencies sharply decrease, necessitating more extensive population data for accurate frequency estimations. UYSD facilitates the merging of data from different studies, including large-scale population studies based on high-throughput sequencing. As WGS costs decline, more population-scale datasets are expected, which will enhance our understanding of high-resolution Y-haplogroups, their origins, and human population history [51]. This also holds forensic relevance for determining paternal biogeographic origins [52]. The examples provided here illustrate how UYSD enables users to easily explore differences in haplogroup frequencies and distributions, across populations and for any Y-haplogroup of interest.

Despite the inclusion of population data from over six thousand males, which were analyzed and uploaded to UYSD, the database remains heavily underpopulated for its goal of providing Y-haplogroup frequencies on a global scale with high geographic coverage. In particular, data from non-European populations are needed to establish a good basis for further expansion. We envision that the platform will gradually grow, with additional datasets from previously published and new Y-haplogroup data. To achieve accurate frequency estimates for the highest-resolution Y-haplogroups, the database will still need to grow by orders of magnitude.

### Conclusions

UYSD was developed to stimulate further exploration of Y chromosome genetic variation, thereby increasing our knowledge of the history of our species and its patterns of migration. With UYSD, we provide the database platform, including a searchable website, that was previously lacking for the scientific and public communities. One of the most important functions is to harmonize data from different studies under a single phylogenetic tree, thereby overcoming difficulties with nomenclature that has changed over the years. Moreover, UYSD is meant to serve as a hub, making it easier for researchers to identify other studies relevant to their own. Lastly, by making UYSD compatible with automated haplogroup prediction from high-throughput sequencing data, it will now become possible to effectively start exploring the deepest branches of the phylogenetic tree.

We encourage authors of new studies to submit their Y-SNP data to UYSD as soon as their paper is published in a peer-reviewed scientific journals. We also encourage authors of Y-SNP studies that were previously published in peer-reviewed scientific journal to submit their data to UYSD. Clearly, the value of the UYSD in providing answers to the various research questions that can be addressed with the help of Y-haplogroup frequency data will grow with each additional dataset included. We hope this study will inspire other scientists to collaborate, allowing us to collectively unlock the full potential of UYSD.

## Supplementary information

Supplementary text

Supplementary Table 1

Supplementary Table 2

Supplementary Figure 1

Supplementary Figure 2

## Data Availability

All data described here can be accessed through https://ysnp.erasmusmc.nl.

## References

[1] Casanova M, Leroy P, Boucekkine C, Weissenbach J, Bishop C, Fellous M, et al. A human Y-linked DNA polymorphism and its potential for estimating genetic and evolutionary distance. Science. 1985;230:1403–6. 2999986 10.1126/science.2999986

[2] Lucotte G, Ngo NY. p49f, A highly polymorphic probe, that detects Taq1 RFLPs on the human Y chromosome. Nucleic Acids Res. 1985;13:8285. 2999718 10.1093/nar/13.22.8285 PMC322126

[3] Hurles ME, Irven C, Nicholson J, Taylor PG, Santos FR, Loughlin J, et al. European Y-chromosomal lineages in Polynesians: a contrast to the population structure revealed by mtDNA. Am J Hum Genet. 1998;63:1793–806. 9837833 10.1086/302147 PMC1377652

[4] Jobling MA, Pandya A, Tyler-Smith C. The Y chromosome in forensic analysis and paternity testing. Int J Leg Med. 1997;110:118–24. 10.1007/s004140050050 9228562

[5] Sanchez JJ, Børsting C, Hallenberg C, Buchard A, Hernandez A, Morling N. Multiplex PCR and minisequencing of SNPs—a model with 35 Y chromosome SNPs. Forensic Sci Int. 2003;137:74–84. 14550618 10.1016/s0379-0738(03)00299-8

[6] Wetton JH, Tsang KW, Khan H. Inferring the population of origin of DNA evidence within the UK by allele-specific hybridization of Y-SNPs. Forensic Sci Int. 2005;152:45–53. 15878814 10.1016/j.forsciint.2005.03.009

[7] Berniell‐Lee G, Sandoval K, Mendizabal I, Bosch E, Comas D. SNPlexing the human Y‐chromosome: A single‐assay system for major haplogroup screening. Electrophoresis. 2007;28:3201–6. 17703471 10.1002/elps.200700078

[8] van Oven M, Ralf A, Kayser M. An efficient multiplex genotyping approach for detecting the major worldwide human Y-chromosome haplogroups. Int J Leg Med. 2011;125:879–85. 10.1007/s00414-011-0605-2 PMC3192277 21785904

[9] Onofri V, Alessandrini F, Turchi C, Pesaresi M, Buscemi L, Tagliabracci A. Development of multiplex PCRs for evolutionary and forensic applications of 37 human Y chromosome SNPs. Forensic Sci Int. 2006;157:23–35. 15896936 10.1016/j.forsciint.2005.03.014

[10] Gomes V, Sánchez-Diz P, Amorim A, Carracedo Á, Gusmão L. Digging deeper into East African human Y chromosome lineages. Hum Genet. 2010;127:603–13. 20213473 10.1007/s00439-010-0808-5

[11] Geppert M, Baeta M, Nunez C, Martínez-Jarreta B, Zweynert S, Cruz OWV, et al. Hierarchical Y-SNP assay to study the hidden diversity and phylogenetic relationship of native populations in South America. Forensic Sci Int: Genet. 2011;5:100–4. 20932815 10.1016/j.fsigen.2010.08.016

[12] van Oven M, Toscani K, van den Tempel N, Ralf A, Kayser M. Multiplex genotyping assays for fine‐resolution subtyping of the major human Y‐chromosome haplogroups E, G, I, J, and R in anthropological, genealogical, and forensic investigations. Electrophoresis. 2013;34:3029–38. 23893838 10.1002/elps.201300210

[13] Karafet TM, Mendez FL, Meilerman MB, Underhill PA, Zegura SL, Hammer MF. New binary polymorphisms reshape and increase resolution of the human Y chromosomal haplogroup tree. Genome Res. 2008;18:830–8. 18385274 10.1101/gr.7172008 PMC2336805

[14] Ralf A, van Oven M, Zhong K, Kayser M. Simultaneous analysis of hundreds of Y‐Chromosomal SNP s for high‐resolution paternal lineage classification using targeted semiconductor sequencing. Hum Mutat. 2015;36:151–9. 25338970 10.1002/humu.22713

[15] Ralf A, van Oven M, González DM, de Knijff P, van der Beek K, Wootton S, et al. Forensic Y-SNP analysis beyond SNaPshot: High-resolution Y-chromosomal haplogrouping from low quality and quantity DNA using Ion AmpliSeq and targeted massively parallel sequencing. Forensic Sci Int Genet. 2019;41:93–106. 31063905 10.1016/j.fsigen.2019.04.001

[16] Tao R, Li M, Chai S, Xia R, Qu Y, Yuan C, et al. Developmental validation of a 381 Y-chromosome SNP panel for haplogroup analysis in the Chinese populations. Forensic Sci Int Genet. 2023;62:102803. 36368220 10.1016/j.fsigen.2022.102803

[17] Tillmar A, Sturk-Andreaggi K, Daniels-Higginbotham J, Thomas JT, Marshall C. The FORCE panel: an all-in-one SNP marker set for confirming investigative genetic genealogy leads and for general forensic applications. Genes. 2021;12:1968. 34946917 10.3390/genes12121968 PMC8702142

[18] Jobling MA, Tyler-Smith C. Human Y-chromosome variation in the genome-sequencing era. Nat Rev Genet. 2017;18:485–97. 28555659 10.1038/nrg.2017.36

[19] Hallast P, Batini C, Zadik D, Maisano Delser P, Wetton JH, Arroyo-Pardo E, et al. The Y-chromosome tree bursts into leaf: 13,000 high-confidence SNPs covering the majority of known clades. Mol Biol Evol. 2015;32:661–73. 25468874 10.1093/molbev/msu327 PMC4327154

[20] Van Geystelen A, Decorte R, Larmuseau MHD. AMY-tree: an algorithm to use whole genome SNP calling for Y chromosomal phylogenetic applications. BMC Genomics. 2013;14:1–12. 23405914 10.1186/1471-2164-14-101 PMC3583733

[21] Poznik GD. Identifying Y-chromosome haplogroups in arbitrarily large samples of sequenced or genotyped men. BioRxiv. 2016. https://www.biorxiv.org/content/10.1101/088716v1.

[22] Ralf A, Montiel González D, Zhong K, Kayser M. Yleaf: software for human Y-chromosomal haplogroup inference from next-generation sequencing data. Mol Biol Evol. 2018;35:1291–4. 29518227 10.1093/molbev/msy032

[23] Yfull. Haplogroup YTree v10.01.00. 2022. Available from: https://www.yfull.com/arch-10.01/tree/.

[24] Van Oven M, Van Geystelen A, Kayser M, Decorte R, Larmuseau MHD. Seeing the wood for the trees: a minimal reference phylogeny for the human Y chromosome. Hum Mutat. 2014;35:187–91. 24166809 10.1002/humu.22468

[25] Bodner M, Bastisch I, Butler JM, Fimmers R, Gill P, Gusmão L, et al. Recommendations of the DNA Commission of the International Society for Forensic Genetics (ISFG) on quality control of autosomal Short Tandem Repeat allele frequency databasing (STRidER). Forensic Sci Int Genet. 2016;24:97–102. 27352221 10.1016/j.fsigen.2016.06.008

[26] Willuweit S, Roewer L, International Forensic YCUG. Y chromosome haplotype reference database (YHRD): update. Forensic Sci Int Genet. 2007;1:83–7. 19083734 10.1016/j.fsigen.2007.01.017

[27] Parson W, Dür A. EMPOP—a forensic mtDNA database. Forensic Sci Int Genet. 2007;1:88–92. 19083735 10.1016/j.fsigen.2007.01.018

[28] Willuweit S, Roewer L. The new Y chromosome haplotype reference database. Forensic Sci Int Genet. 2015;15:43–8. 25529991 10.1016/j.fsigen.2014.11.024

[29] Django Software Foundation. Django. 2013. Available from: https://www.djangoproject.com/.

[30] Van Rossum G, Drake FL. Python reference manual: Centrum voor Wiskunde en Informatica Amsterdam; 1995.

[31] Hipp RD SQLite. 2020. Available from: https://www.sqlite.org/index.html.

[32] Agafonkin V. Leaflet, an open-source JavaScript library for mobile-friendly interactive maps. 2010. Available from: https://leafletjs.com/.

[33] OpenStreetMap contributors. 2017. Available from: https://www.openstreetmap.org.

[34] MapTiles API. 2025. Available from: https://www.maptilesapi.com/.

[35] Natural Earth. 2009. Available from: https://www.naturalearthdata.com/.

[36] Excoffier L, Lischer HEL. Arlequin suite ver 3.5: a new series of programs to perform population genetics analyses under Linux and Windows. Mol Ecol Resour. 2010;10:564–7. 21565059 10.1111/j.1755-0998.2010.02847.x

[37] Nei M. Analysis of gene diversity in subdivided populations. Proc Natl Acad Sci. 1973;70:3321–3. 4519626 10.1073/pnas.70.12.3321 PMC427228

[38] Chiaroni J, Underhill PA, Cavalli-Sforza LL. Y chromosome diversity, human expansion, drift, and cultural evolution. Proc Natl Acad Sci. 2009;106:20174–9. 19920170 10.1073/pnas.0910803106 PMC2787129

[39] Myres NM, Rootsi S, Lin AA, Järve M, King RJ, Kutuev I, et al. A major Y-chromosome haplogroup R1b Holocene era founder effect in Central and Western Europe. Eur J Hum Genet. 2011;19:95–101. 20736979 10.1038/ejhg.2010.146 PMC3039512

[40] Underhill PA, Poznik GD, Rootsi S, Järve M, Lin AA, Wang J, et al. The phylogenetic and geographic structure of Y-chromosome haplogroup R1a. Eur J Hum Genet. 2015;23:124–31. 24667786 10.1038/ejhg.2014.50 PMC4266736

[41] Rootsi S, Kivisild T, Benuzzi G, Bermisheva M, Kutuev I, Barać L, et al. Phylogeography of Y-chromosome haplogroup I reveals distinct domains of prehistoric gene flow in Europe. Am J Hum Genet. 2004;75:128–37. 15162323 10.1086/422196 PMC1181996

[42] Semino O, Magri C, Benuzzi G, Lin AA, Al-Zahery N, Battaglia V, et al. Origin, diffusion, and differentiation of Y-chromosome haplogroups E and J: inferences on the neolithization of Europe and later migratory events in the Mediterranean area. Am J Hum Genet. 2004;74:1023–34. 15069642 10.1086/386295 PMC1181965

[43] Battaglia V, Grugni V, Perego UA, Angerhofer N, Gomez-Palmieri JE, Woodward SR, et al. The first peopling of South America: new evidence from Y-chromosome haplogroup Q. PLoS One. 2013;8:e71390. 23990949 10.1371/journal.pone.0071390 PMC3749222

[44] Cruciani F, La Fratta R, Trombetta B, Santolamazza P, Sellitto D, Colomb EB, et al. Tracing past human male movements in northern/eastern Africa and western Eurasia: new clues from Y-chromosomal haplogroups E-M78 and J-M12. Mol Biol Evol. 2007;24:1300–11. 17351267 10.1093/molbev/msm049

[45] Larmuseau MHD, Otten GPPL, Decorte R, Van Damme P, Moisse M. Defining Y-SNP variation among the Flemish population (Western Europe) by full genome sequencing. Forensic Sci Int Genet. 2017;31:e12–e6. 29089250 10.1016/j.fsigen.2017.10.008

[46] Ameur A, Dahlberg J, Olason P, Vezzi F, Karlsson R, Martin M, et al. SweGen: a whole-genome data resource of genetic variability in a cross-section of the Swedish population. Eur J Hum Genet. 2017;25:1253–60. 28832569 10.1038/ejhg.2017.130 PMC5765326

[47] Otagiri T, Sato N, Asamura H, Parvanova E, Kayser M, Ralf A. RMplex reveals population differences in RM Y-STR mutation rates and provides improved father-son differentiation in Japanese. Forensic Sci Int Genet. 2022;61:102766. 36007266 10.1016/j.fsigen.2022.102766

[48] Ottoni C, Larmuseau MHD, Vanderheyden N, Martínez‐Labarga C, Primativo G, Biondi G, et al. Deep into the roots of the Libyan Tuareg: a genetic survey of their paternal heritage. Am J Phys Anthropol. 2011;145:118–24. 21312181 10.1002/ajpa.21473

[49] Larmuseau MHD, Vessi A, Jobling MA, Van Geystelen A, Primativo G, Biondi G, et al. The paternal landscape along the Bight of Benin–Testing regional representativeness of West-African population samples using Y-chromosomal markers. PLoS One. 2015;10:e0141510. 26544036 10.1371/journal.pone.0141510 PMC4636292

[50] Rootsi S, Zhivotovsky LA, Baldovič M, Kayser M, Kutuev IA, Khusainova R, et al. A counter-clockwise northern route of the Y-chromosome haplogroup N from Southeast Asia towards Europe. Eur J Hum Genet. 2007;15:204–11. 17149388 10.1038/sj.ejhg.5201748

[51] Hallast P, Ebert P, Loftus M, Yilmaz F, Audano PA, Logsdon GA, et al. Assembly of 43 human Y chromosomes reveals extensive complexity and variation. Nature. 2023;621:355–64. 37612510 10.1038/s41586-023-06425-6 PMC10726138

[52] Kayser M. Forensic use of Y-chromosome DNA: a general overview. Hum Genet. 2017;136:621–35. 28315050 10.1007/s00439-017-1776-9 PMC5418305

