# Supplementary material for: UYSD: a novel data repository accessible via public website for worldwide population frequencies of Y-SNP haplogroups
Source: Eur J Hum Genet. 2025 May 8;33(7):904–12. doi: 10.1038/s41431-025-01854-5 (PMC12229683; doi:10.1038/s41431-025-01854-5)
Supplement: Supplementary file 1 — Supplementary text [file 41431_2025_1854_MOESM1_ESM.pdf]

UYSD

User Manual v1.1

2025

Erasmus MC

Contents

1. Introduction 3
2. YSNP database 4
3. Sample database 4
4. Haplogroup map 5
   4.1 Performing searches 5
   4.2 Display options 5
   4.3 Analyzing the results 6
5. Haplogroup tree 6
6. Data submission 7
   6.1 Submission Methods 7
   6.2 Sub-Country Region Information 8
7. About 8

1. Introduction

The Universal Y-SNP Database (UYSD) is an interactive online platform designed to facilitate the study of human Y-chromosome genetic variation. By harmonizing diverse datasets under a unified phylogenetic tree, UYSD provides researchers and the public with a comprehensive resource for exploring Y-SNP and haplogroup distributions across global populations.

Scientific interest in paternal lineages has grown over the decades, but relevant data has often been fragmented across numerous publications, making it difficult to consolidate and analyze effectively. UYSD addresses this challenge by offering a searchable and accessible database that standardizes haplogroup information, ensuring consistency in nomenclature despite historical changes.

The platform enables users to:

- Search and visualize Y-haplogroup distributions on an interactive world map.
- Estimate haplogroup frequencies in regions with sparse data using interpolation.
- Access and explore a detailed phylogenetic tree of Y-chromosomal haplogroups.
- Submit and integrate new datasets, fostering collaboration and data sharing within the scientific community.

UYSD is compatible with both high- and low-throughput sequencing technologies and supports automated haplogroup prediction via Yleaf v3. This functionality allows researchers to analyze the deepest branches of the Y-chromosome phylogenetic tree more effectively.

By continuously expanding and refining its dataset, UYSD serves as a valuable tool for researchers in fields such as human population genetics, genetic anthropology, forensic genetics, and ancient DNA analysis. The platform encourages authors of newly published and previously published Y-SNP studies to contribute their data, helping to enhance the collective understanding of human migration patterns and genetic diversity.

This manual provides detailed guidance on how to navigate and utilize UYSD, including data submission, searching, and visualization features.

2. YSNP database

This section of the website serves as a resource for finding Y-SNPs of interest. It includes a database containing all Y-SNPs incorporated into UYSD, allowing users to search for specific Y-SNPs and access their associated information simply by typing the name of the Y-SNP of interest.

Alternatively, users can search for a specific haplogroup to retrieve all Y-SNPs (including alternative names and equivalent Y-SNPs) that define that haplogroup. It is also possible to search within a specific genomic region of hg19 (GRCh37) or hg38 (GRCh38) to display all included Y-SNPs within that region. While the majority of Y-SNPs do not have an associated rs number, users can search for those that are included in the database.

Additionally, this section of the website provides seamless navigation to related features based on the selected haplogroup. With a single click, users can access the haplogroup map, sample list, or locate the haplogroup in the phylogenetic tree utilized by UYSD.

3. Sample database

All samples included in UYSD are available in the Sample Database (without displaying sample IDs). This page provides additional details about each sample, including the date of submission, the contributor’s name, and, if analyzed using Yleaf, the corresponding quality control (QC) score.

Additionally, the page includes a feature that allows users to directly contact the individual who submitted the data through a built-in contact form. This facilitates collaboration and enables researchers to seek further information about specific datasets.

The sample database supports multiple search criteria, allowing users to filter results based on haplogroup, author, country, or region. When searching for a country without specifying a region, a colon (:) must be added after the country name to ensure accurate results. For example, to search for all samples from Belgium without regional details, enter "Belgium:" in the search bar. When searching specifically for data from the region of East Flanders type “Belgium:East Flanders” instead.

4. Haplogroup map

This page features one of the core functionalities of UYSD—generating interactive geographic maps that display the frequency of a given haplogroup in populations where it has been analyzed.

4.1 Performing searches

Users can search for a haplogroup or SNP by simply typing its name into the search bar. Searches are not case-sensitive. The percentages displayed on the map represent the proportion of samples typed for the queried haplogroup. This percentage is calculated as: (Number of samples with the derived allele) / (Total samples with either the derived or ancestral allele). Results are generated only if at least one Y-SNP defining the searched haplogroup is present in the analyzed data. Consequently, the number of countries which show frequency data and the number of samples per country may vary depending on the search performed.

In addition to single haplogroup searches, users can refine results using the asterisk (*) notation. This feature helps identify samples that are derived for a primary haplogroup but ancestral for specified subclades. For example, searching for R1b*(xL51) will return all samples that are derived for R1b but ancestral for R-L51.

4.2 Display options

Interpolation feature (Use interpolation checkbox)
This feature estimates haplogroup frequencies in regions without available data by analyzing surrounding areas. For a region to be eligible for interpolation, it must have at least three neighboring regions with known frequencies within 1,000 kilometers (of the borders). The estimated frequency is calculated using a weighted average, where closer regions have a stronger influence on the final value. When this feature is enabled, hovering over a country will display either 'Interpolation: false,' meaning the frequency estimate is based on actual population data, or 'Interpolation: true,' indicating that the estimate was derived using the interpolation function.

Country vs. Regional View (Only Show Country checkbox)
When enabled, the map displays haplogroup frequencies for entire countries. When disabled and if sub-country regional data is available, the map will show individual regional frequencies instead.

Dynamic vs. Absolute Scaling (Dynamic Colors checkbox)

Dynamic Scaling: Adjusts the color gradient to reflect the highest observed frequency in the dataset. If unchecked a fixed scale ranging from 0% to 100% is used for consistent comparisons.

4.3 Analyzing the results

Once a search has been performed, a world map will be displayed, where colors indicate the regions for which haplogroup frequency data is available. By hovering over a country or region with the mouse, users can view the sample size and the observed frequency of the haplogroup in that area.

At the top of the page, the phylogenetic pathway leading to the searched haplogroup is shown. This includes the parent haplogroups as well as any direct subclades that further subdivide the searched haplogroup. Each haplogroup in this pathway is clickable, allowing users to quickly navigate to the map for that specific haplogroup.

In the bottom-right corner of the map, a color scale is displayed, indicating the frequency range in which the haplogroup was observed.

Viewing Country-Specific Data
If a country has a non-zero frequency, users can click on it to access detailed sample data in table format. For example, when searching for R1b, the table may include numerous samples from various sub-branches of the R1b clade.

The table provides additional information, including:

- The author(s) who submitted the data matching the search criteria.
- A link to the publication associated with the dataset.
- An option to contact the submitter directly through a built-in form.

Exploring Phylogenetic Context
After selecting a country, an option labeled “Show all haplogroups for this country in a tree” will appear above the map. Clicking this link navigates to a page displaying all samples from the selected country in their full phylogenetic context, allowing for a deeper exploration of the haplogroup structure within that region.

5. Haplogroup tree

Phylogenetic Y-haplogroup trees have grown increasingly extensive. To help users keep track of the haplogroups included in UYSD and understand their relationships, the platform provides an

interactive tree view. This feature allows users to explore the entire tree and search for specific haplogroups to determine their phylogenetic positions.

The tree is interactive—by clicking on a haplogroup the downstream phylogenetic path will be highlighted in red. Additionally after clicking a haplogroup and using the hyperlinks in the panel on the right side of the screen, users can:

- Navigate to its geographic frequency map.
- View a list of associated Y-SNPs.
- Access the samples in UYSD that belong to this haplogroup.

This functionality enables seamless exploration of haplogroup relationships and their global distribution.

6. Data submission

The Data Submission page is accessible only to registered users of UYSD. To gain access, users must be affiliated with an academic institution. Once an account has been created, contributors can view or delete the samples they have provided at any time.

Submitting data requires agreeing to the Terms and Conditions, which are available on the Data Submission page.

6.1 Submission Methods

There are two ways to submit data:

- Yleaf Output Files:

  The easiest and most streamlined method for uploading data.

- Manual Submission:

  Upload a list of Y-SNPs typed in an assay, along with a file specifying the final haplogroup assignment for each sample.

For both submission methods, an additional file is required containing:

- Sample IDs (these will not be displayed within UYSD) (required).

- Country (required).
- Sub-country region (optional).
- Additional comments about the samples to be displayed on UYSD (optional).
- A link to the original publication where the samples were first described (required).

6.2 Sub-Country Region Information

The Data Submission page also offers an option to view all sub-country regions that can be included in UYSD. This information is available in two formats:

- Interactive world map.
- Detailed list view.

7. About

The About page contains a contact form which can be used to contact the administrator of UYSD in case of questions or concerns. It also contains this manual and a link to the publication of UYSD.
