# Supplementary material for: UYSD: a novel data repository accessible via public website for worldwide population frequencies of Y-SNP haplogroups
Source: Eur J Hum Genet. 2025 May 8;33(7):904–12. doi: 10.1038/s41431-025-01854-5 (PMC12229683; doi:10.1038/s41431-025-01854-5)
Supplement: Supplementary file 2 — Supplementary Table 1 [file 41431_2025_1854_MOESM2_ESM.pdf]

**Population**	Albania
**Responsible entity**	Università degli Studi di Modena e Reggio Emilia
**No. of participants**	188
**Level of resolution**	Ampliseq panel
**Informed consent**	N.a.
**Ethical approval no.**	N.a.
**Year of sample collection**	2010
**Original publication**	N.a.
**Other comments**	N.a.

**Population**	Austria
**Responsible entity**	University of Salzburg
**No. of participants**	380
**Level of resolution**	Ampliseq panel
**Informed consent**	N.a.
**Ethical approval no.**	N.a.
**Year of sample collection**	2005-2015
**Original publication**	N.a.
**Other comments**	N.a.

**Population**	Belgium
**Responsible entity**	KU Leuven
**No. of participants**	270
**Level of resolution**	WGS
**Informed consent**	Yes
**Ethical approval no.**	Medical Ethical Committee - KU Leuven, nr. S50354
**Year of sample collection**	2014
**Original publication**	https://doi.org/10.1016/j.fsigen.2017.10.008
**Other comments**	N.a.

**Population**	Benin
**Responsible entity**	University of Madeira
**No. of participants**	169
**Level of resolution**	Ampliseq panel
**Informed consent**	Yes
**Ethical approval no.**	CER-ISBA nº 128, 18/05/2020
**Year of sample collection**	2020
**Original publication**	N.a.
**Other comments**	N.a.

**Population**	Benin
**Responsible entity**	KU Leuven
**No. of participants**	120
**Level of resolution**	SNaPshot assay
**Informed consent**	Yes
**Ethical approval no.**	Comitato Etico Indipendente; decision on 14 June 2011 for the “Protocollo di Studio Benin" (submitted by Olga Rickards)
**Year of sample collection**	1989
**Original publication**	https://doi.org/10.1371/journal.pone.0141510
**Other comments**	N.a.

**Population**	Croatia
**Responsible entity**	Institute for Anthropological Research
**No. of participants**	185
**Level of resolution**	Ampliseq panel
**Informed consent**	N.a.
**Ethical approval no.**	N.a.
**Year of sample collection**	2000-2017
**Original publication**	N.a.
**Other comments**	N.a.

**Population**	Czech Republic
**Responsible entity**	Institute of Criminalistics, Czech Republic
**No. of participants**	182
**Level of resolution**	Ampliseq panel
**Informed consent**	Yes
**Ethical approval no.**	N.a.
**Year of sample collection**	2015-2019
**Original publication**	N.a.
**Other comments**	Czech unrelated samples have been collected for the past 15 years among volunteers, with their general signed informed consent

**Population**	Estonia
**Responsible entity**	University of Tartu
**No. of participants**	400
**Level of resolution**	WGS
**Informed consent**	Yes
**Ethical approval no.**	EBIN_1.1-12/1346 (03/07/2020)
**Year of sample collection**	2000-2010
**Original publication**	N.a.
**Other comments**	Data was generated using WGS but genotypes for a panel of 770 selected informative positions were submitted.

**Population**	Germany
**Responsible entity**	University of Cologne
**No. of participants**	189
**Level of resolution**	Ampliseq panel
**Informed consent**	Yes
**Ethical approval no.**	N.a.
**Year of sample collection**	2010-2019
**Original publication**	N.a.
**Other comments**	All samples origin from paternity cases. For all samples we have informed consent for the scientific use in anonymized form. This is regulated by the Genetic Diagnosis Act - GenDG §13 (2). As the samples are anonymized, we of course cant go back to the informed.consent

**Population**	Greece
**Responsible entity**	Aristotle University of Thessaloniki
**No. of participants**	187
**Level of resolution**	Ampliseq panel
**Informed consent**	Yes
**Ethical approval no.**	N.a.
**Year of sample collection**	2002-2003
**Original publication**	N.a.
**Other comments**	N.a.
**Responsible entity**	Hungarian Institute for Forensic Sciences, Institute of Forensic Genetics, Budapest Hungary

**Population**	Hungary
**Responsible entity**	Hungarian Institute for Forensic Sciences, Institute of Forensic Genetics, Budapest Hungary
**No. of participants**	191
**Level of resolution**	Ampliseq panel
**Informed consent**	N.a.
**Ethical approval no.**	N.a.
**Year of sample collection**	2004-2014
**Original publication**	N.a.
**Other comments**	N.a.

**Population**	Iraq
**Responsible entity**	University of Cologne
**No. of participants**	192
**Level of resolution**	Ampliseq panel
**Informed consent**	Yes
**Ethical approval no.**	N.a.
**Year of sample collection**	2014-2019
**Original publication**	N.a.
**Other comments**	All samples origin from paternity cases. For all samples we have informed consent for the scientific use in anonymized form. This is regulated by the Genetic Diagnosis Act - GenDG §13 (2). As the samples are anonymized, we of course cant go back to the informed.consent

**Population**	Italy
**Responsible entity**	Università degli Studi di Modena e Reggio Emilia
**No. of participants**	186
**Level of resolution**	Ampliseq panel
**Informed consent**	N.a.
**Ethical approval no.**	N.a.
**Year of sample collection**	2008
**Original publication**	N.a.
**Other comments**	N.a.

**Population**	Kazakhstan
**Responsible entity**	Nazarbayev University
**No. of participants**	185
**Level of resolution**	Ampliseq panel
**Informed consent**	Yes
**Ethical approval no.**	1. 13 March 2019; 2. №2, 10 June 2020;
**Year of sample collection**	2019
**Original publication**	N.a.
**Other comments**	Confidentiality & Privacy. We will store participant's signed consent form in a locked file; only members of the study team will have an access to this file.

**Population**	Lesotho
**Responsible entity**	University of the Western Cape
**No. of participants**	93
**Level of resolution**	Ampliseq panel
**Informed consent**	Yes
**Ethical approval no.**	Ministry of Health, Lesotho (ID128-20016); export permits were approved by the Lesotho Ministry of Health.
**Year of sample collection**	2017-2018
**Original publication**	https://doi.org/10.1016/j.fsigss.2019.10.033
**Other comments**	N.a.

**Population**	Libya
**Responsible entity**	KU Leuven
**No. of participants**	47
**Level of resolution**	SNaPshot assay
**Informed consent**	Yes
**Ethical approval no.**	Historical collection: Italian-Libyan Archaeological Mission in the Acacus and Messak (Libyan Sahara) of the University of Rome, La Sapienza, and the Department of Antiquities, Tripoli, directed by Prof. Savino di Lernia and the late Ebrahim Azzebi
**Year of sample collection**	2003
**Original publication**	https://doi.org/10.1002/ajpa.21473
**Other comments**	N.a.

**Population**	Mexico
**Responsible entity**	University of Guadalajara
**No. of participants**	169
**Level of resolution**	Ampliseq panel
**Informed consent**	N.a.
**Ethical approval no.**	33949
**Year of sample collection**	2018-2019
**Original publication**	N.a.
**Other comments**	Confidentiality & Privacy. We will store participant's signed consent form in a locked file; only members of the study team will have an access to this file.

**Population**	Netherlands
**Responsible entity**	Erasmus MC University Medical Center Rotterdam
**No. of participants**	192
**Level of resolution**	Ampliseq panel
**Informed consent**	Yes
**Ethical approval no.**	MEC-2020-0240
**Year of sample collection**	Before 2010
**Original publication**	https://doi.org/10.1038/ejhg.2013.118
**Other comments**	N.a.

**Population**	Poland
**Responsible entity**	Medical University of Gdansk
**No. of participants**	187
**Level of resolution**	Ampliseq panel
**Informed consent**	Yes
**Ethical approval no.**	NKEBN/199/2009
**Year of sample collection**	2009
**Original publication**	N.a.
**Other comments**	N.a.

**Population**	Portugal
**Responsible entity**	University of Madeira
**No. of participants**	191
**Level of resolution**	Ampliseq panel
**Informed consent**	No
**Ethical approval no.**	N.a.
**Year of sample collection**	1999-2000
**Original publication**	N.a.
**Other comments**	Portuguese samples were collected more than 20 years ago among army volunteers, with the agreement of the Chief of the Armed forces. There are no signed informed consents or appproval of ethical committe,

**Population**	Slovakia
**Responsible entity**	Institute of Forensic Science, Slovak Republic
**No. of participants**	192
**Level of resolution**	Ampliseq panel
**Informed consent**	Yes
**Ethical approval no.**	N.a.
**Year of sample collection**	2020-2021
**Original publication**	N.a.
**Other comments**	N.a.

**Population**	South Africa
**Responsible entity**	University of the Western Cape
**No. of participants**	96
**Level of resolution**	Ampliseq panel
**Informed consent**	Yes
**Ethical approval no.**	University of the Western Cape Senate Ethics and Research Committee (SR 01/3/10, SR 15–4–97, BM/16/3/18); University of Limpopo (TREC/09/2016:R); approval for collection at the University of Venda and University of the Frees State; export permits were approved by the South African Department of Health and the Lesotho Ministry of Health.
**Year of sample collection**	2011-2018
**Original publication**	https://doi.org/10.1016/j.fsigen.2022.102677
**Other comments**	N.a.

**Population**	Sweden
**Responsible entity**	Uppsala University
**No. of participants**	475
**Level of resolution**	WGS
**Informed consent**	Yes
**Ethical approval no.**	Regionala Etikprövningsnämnden, Stockholm, dnr 2007-644-31, dnr 2014/521-32
**Year of sample collection**	2004-2008
**Original publication**	https://doi.org/10.1038/ejhg.2017.130
**Other comments**	N.a.

**Population**	Switzerland
**Responsible entity**	University of Zurich
**No. of participants**	189
**Level of resolution**	Ampliseq panel
**Informed consent**	Yes
**Ethical approval no.**	case number 2022-08
**Year of sample collection**	2004, 2010, and 2020
**Original publication**	N.a.
**Other comments**	Evaluated by the CEBES review board at the University of Zurich (now called Ethics commission of the Medical Faculty)

**Population**	United Kingdom
**Responsible entity**	University of Leicester
**No. of participants**	190
**Level of resolution**	Ampliseq panel
**Informed consent**	Yes
**Ethical approval no.**	N.a.
**Year of sample collection**	2000-2005
**Original publication**	http://dx.doi.org/10.1016/j.fsigen.2014.04.008
**Other comments**	N.a.

**Population**	United States of America
**Responsible entity**	National Institute of Standards and Technology
**No. of participants**	1050
**Level of resolution**	Ampliseq panel
**Informed consent**	N.a.
**Ethical approval no.**	MML-16-0080
**Year of sample collection**	2002/2005
**Original publication**	https://doi.org/10.1111/j.1556-4029.2007.00491.x
**Other comments**	All work has been reviewed and approved by the National Institute of Standards and Technology Research Protections Office. This study was determined to be "not human subjects research" (often referred to as research not involving human subjects) as defined in U. S. Code of Federal Regulations, 15 CFR 27, also known as the Common Rule (45 CFR 46, Subpart A), for the Protection of Human Subjects by the NIST Human Research Protections Office and therefore not subject to oversight by the NIST Institutional Review Board.

**Population**	Japan
**Responsible entity**	National Research Institute of Police Science Japan
**No. of participants**	192
**Level of resolution**	Ampliseq panel
**Informed consent**	Yes
**Ethical approval no.**	32-1(74)
**Year of sample collection**	2001-2006
**Original publication**	N.a.
**Other comments**	N.a.

**Population**	Japan
**Responsible entity**	Erasmus MC University Medical Center Rotterdam
**No. of participants**	161
**Level of resolution**	SNaPshot assay
**Informed consent**	Yes
**Ethical approval no.**	Shinshu University School of Medicine (Permission number: 667)
**Year of sample collection**	Before 2021
**Original publication**	https://doi.org/10.1016/j.fsigen.2022.102766
**Other comments**	N.a.

**Population**	Philippines
**Responsible entity**	University of the Philippines Diliman
**No. of participants**	189
**Level of resolution**	Ampliseq panel
**Informed consent**	Yes
**Ethical approval no.**	University of the Philippines Manila Research Ethics Board (UPMREB 2017-090-01) for Secondary Use; UPMREB 2012-247-01 and 2012-0276 for sample collection
**Year of sample collection**	2013
**Original publication**	N.a.
**Other comments**	N.a.
