# Supplementary material for: UYSD: a novel data repository accessible via public website for worldwide population frequencies of Y-SNP haplogroups
Source: Eur J Hum Genet. 2025 May 8;33(7):904–12. doi: 10.1038/s41431-025-01854-5 (PMC12229683; doi:10.1038/s41431-025-01854-5)
Supplement: Supplementary file 3 — Supplementary Table 2 [file 41431_2025_1854_MOESM3_ESM.pdf]

Computing conventional F-Statistics from haplotype frequencies using Arlequin v3.5

	Albania	Austria	Belgium	Benin	Croatia	Czech Republic	Germany	Greece	Hungary	Iraq	Italy	Japan	Kazakhstan	Lesotho	Libya	Mexico	Netherlands	Philippines	Poland	Portugal	Slovakia	South Africa	Sweden	Switzerland	United Kingdom	United States of America
**Albania**	0.000	0.045	0.073	0.161	0.072	0.063	0.059	0.022	0.054	0.064	0.071	0.121	0.195	0.143	0.249	0.117	0.078	0.153	0.118	0.112	0.084	0.144	0.083	0.069	0.133	0.070
**Austria**	0.045	0.000	0.014	0.109	0.047	0.020	0.003	0.011	0.016	0.022	0.023	0.071	0.140	0.090	0.191	0.063	0.011	0.101	0.061	0.057	0.035	0.091	0.027	0.008	0.073	0.018
**Belgium**	0.073	0.014	0.000	0.116	0.084	0.041	0.007	0.032	0.038	0.033	0.020	0.077	0.150	0.095	0.199	0.056	0.005	0.108	0.084	0.034	0.065	0.094	0.027	0.003	0.040	0.009
**Benin**	0.161	0.109	0.116	0.000	0.170	0.133	0.112	0.119	0.129	0.115	0.132	0.150	0.225	0.028	0.276	0.154	0.115	0.184	0.166	0.159	0.150	0.028	0.116	0.117	0.174	0.073
**Croatia**	0.072	0.047	0.084	0.170	0.000	0.041	0.054	0.047	0.018	0.082	0.091	0.129	0.199	0.153	0.259	0.127	0.085	0.163	0.072	0.125	0.020	0.154	0.086	0.080	0.145	0.079
**Czech Republic**	0.063	0.020	0.041	0.133	0.041	0.000	0.022	0.028	0.012	0.047	0.052	0.093	0.165	0.113	0.218	0.088	0.041	0.125	0.012	0.086	0.008	0.114	0.048	0.035	0.102	0.042
**Germany**	0.059	0.003	0.007	0.112	0.054	0.022	0.000	0.018	0.020	0.025	0.022	0.073	0.147	0.092	0.194	0.060	0.005	0.105	0.061	0.047	0.039	0.092	0.023	0.006	0.068	0.014
**Greece**	0.022	0.011	0.032	0.119	0.047	0.028	0.018	0.000	0.022	0.019	0.033	0.082	0.154	0.098	0.207	0.072	0.034	0.112	0.074	0.067	0.047	0.099	0.044	0.029	0.094	0.031
**Hungary**	0.054	0.016	0.038	0.129	0.018	0.012	0.020	0.022	0.000	0.042	0.047	0.088	0.159	0.108	0.211	0.083	0.038	0.120	0.035	0.077	0.008	0.109	0.045	0.032	0.097	0.037
**Iraq**	0.064	0.022	0.033	0.115	0.082	0.047	0.025	0.019	0.042	0.000	0.041	0.074	0.146	0.094	0.194	0.070	0.033	0.106	0.086	0.074	0.064	0.095	0.040	0.033	0.094	0.031
**Italy**	0.071	0.023	0.020	0.132	0.091	0.052	0.022	0.033	0.047	0.041	0.000	0.092	0.167	0.111	0.215	0.070	0.034	0.124	0.100	0.036	0.076	0.111	0.049	0.014	0.079	0.028
**Japan**	0.121	0.071	0.077	0.150	0.129	0.093	0.073	0.082	0.088	0.074	0.092	0.000	0.181	0.132	0.233	0.115	0.076	0.124	0.126	0.120	0.110	0.133	0.078	0.077	0.133	0.063
**Kazakhstan**	0.195	0.140	0.150	0.225	0.199	0.165	0.147	0.154	0.159	0.146	0.167	0.181	0.000	0.215	0.324	0.192	0.151	0.216	0.198	0.197	0.178	0.216	0.147	0.152	0.211	0.137
**Lesotho**	0.143	0.090	0.095	0.028	0.153	0.113	0.092	0.098	0.108	0.094	0.111	0.132	0.215	0.000	0.273	0.135	0.094	0.168	0.149	0.138	0.131	-0.007	0.097	0.096	0.157	0.058
**Libya**	0.249	0.191	0.199	0.276	0.259	0.218	0.194	0.207	0.211	0.194	0.215	0.233	0.324	0.273	0.000	0.234	0.198	0.274	0.255	0.227	0.236	0.273	0.197	0.202	0.263	0.182
**Mexico**	0.117	0.063	0.056	0.154	0.127	0.088	0.060	0.072	0.083	0.070	0.070	0.115	0.192	0.135	0.234	0.000	0.064	0.148	0.128	0.076	0.109	0.135	0.075	0.058	0.106	0.046
**Netherlands**	0.078	0.011	0.005	0.115	0.085	0.041	0.005	0.034	0.038	0.033	0.034	0.076	0.151	0.094	0.198	0.064	0.000	0.107	0.082	0.056	0.064	0.094	0.024	0.008	0.063	0.015
**Philippines**	0.153	0.101	0.108	0.184	0.163	0.125	0.105	0.112	0.120	0.106	0.124	0.124	0.216	0.168	0.274	0.148	0.107	0.000	0.159	0.154	0.142	0.169	0.108	0.109	0.167	0.096
**Poland**	0.118	0.061	0.084	0.166	0.072	0.012	0.061	0.074	0.035	0.086	0.100	0.126	0.198	0.149	0.255	0.128	0.082	0.159	0.000	0.130	0.017	0.150	0.079	0.079	0.144	0.078
**Portugal**	0.112	0.057	0.034	0.159	0.125	0.086	0.047	0.067	0.077	0.074	0.036	0.120	0.197	0.138	0.227	0.076	0.056	0.154	0.130	0.000	0.108	0.139	0.074	0.034	0.094	0.044
**Slovakia**	0.084	0.035	0.065	0.150	0.020	0.008	0.039	0.047	0.008	0.064	0.076	0.110	0.178	0.131	0.236	0.109	0.064	0.142	0.017	0.108	0.000	0.132	0.065	0.060	0.127	0.061
**South Africa**	0.144	0.091	0.094	0.028	0.154	0.114	0.092	0.099	0.109	0.095	0.111	0.133	0.216	-0.007	0.273	0.135	0.094	0.169	0.150	0.139	0.132	0.000	0.097	0.096	0.151	0.057
**Sweden**	0.083	0.027	0.027	0.116	0.086	0.048	0.023	0.044	0.045	0.040	0.049	0.078	0.147	0.097	0.197	0.075	0.024	0.108	0.079	0.074	0.065	0.097	0.000	0.027	0.079	0.028
**Switzerland**	0.069	0.008	0.003	0.117	0.080	0.035	0.006	0.029	0.032	0.033	0.014	0.077	0.152	0.096	0.202	0.058	0.008	0.109	0.079	0.034	0.060	0.096	0.027	0.000	0.061	0.014
**United Kingdom**	0.133	0.073	0.040	0.174	0.145	0.102	0.068	0.094	0.097	0.094	0.079	0.133	0.211	0.157	0.263	0.106	0.063	0.167	0.144	0.094	0.127	0.151	0.079	0.061	0.000	0.042
**United States of America**	0.070	0.018	0.009	0.073	0.079	0.042	0.014	0.031	0.037	0.031	0.028	0.063	0.137	0.058	0.182	0.046	0.015	0.096	0.078	0.044	0.061	0.057	0.028	0.014	0.042	0.000
