# Supplementary figures and images for: UYSD: a novel data repository accessible via public website for worldwide population frequencies of Y-SNP haplogroups
Source: Eur J Hum Genet. 2025 May 8;33(7):904–12. doi: 10.1038/s41431-025-01854-5 (PMC12229683; doi:10.1038/s41431-025-01854-5)

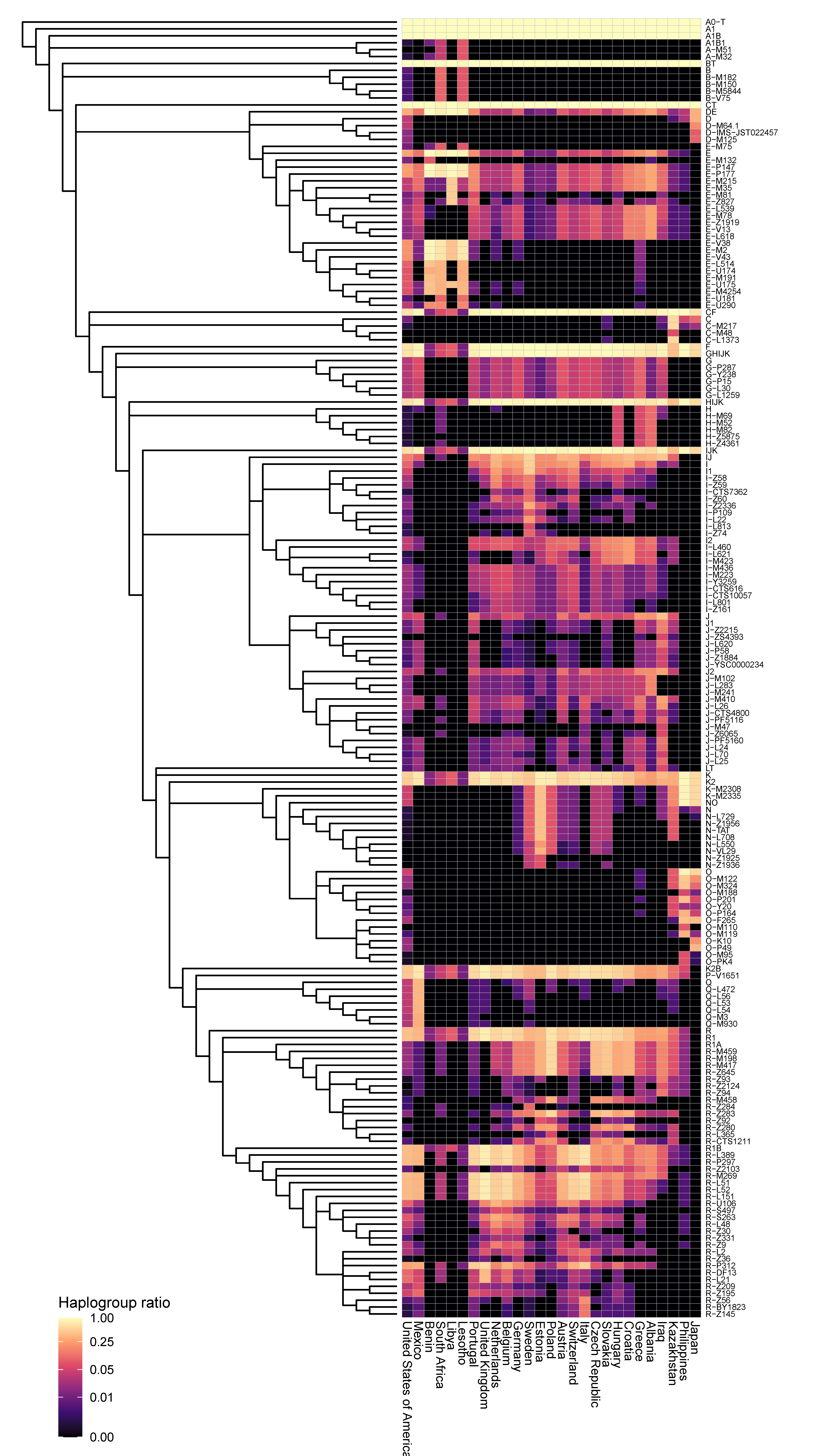

Supplement: Supplementary file 4 — Supplementary Figure 1 [file 41431_2025_1854_MOESM4_ESM.tif]

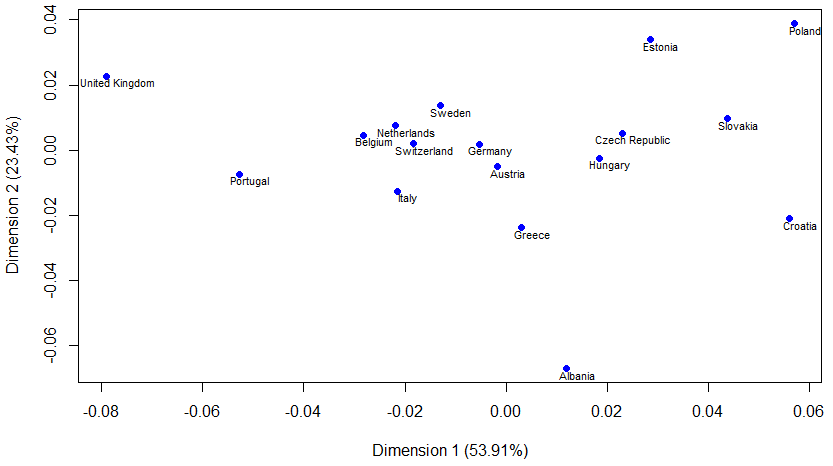

Supplement: Supplementary file 5 — Supplementary Figure 2 [file 41431_2025_1854_MOESM5_ESM.tif]
